# Three new species of *Cantharellales* and *Hymenochaetales* from *Pinus massoniana* in China

**DOI:** 10.3897/mycokeys.137.203862

**Published:** 2026-07-24

**Authors:** Kai-Yue Luo, Yu-Cheng Dai, Yuan Yuan, Ying-Da Wu

**Affiliations:** 1 State Key Laboratory of Efficient Production of Forest Resources, School of Ecology and Nature Conservation, Beijing Forestry University, Beijing 100083, China Key Laboratory of Ministry of Emergency Management for Forest and Grassland Fire Risk Prevention, China Fire and Rescue Institute Beijing China; 2 Key Laboratory of Ministry of Emergency Management for Forest and Grassland Fire Risk Prevention, China Fire and Rescue Institute, Beijing 102202, China State Key Laboratory of Efficient Production of Forest Resources, School of Ecology and Nature Conservation, Beijing Forestry University Beijing China

**Keywords:** Corticioid fungi, molecular phylogeny, taxonomy, wood-inhabiting fungi

## Abstract

Three new wood-inhabiting fungi species, found on *Pinus massoniana*, are proposed, based on a combination of morphological features and molecular evidence. *Botryobasidium massonianae*, belonging to *Cantharellales*, is characterised by fluffy, pellicular basidiomata, hypochnoid hymenophoral surface, generative hyphae simple septate, basidia with four to six sterigmata and basidiospores measuring 6.3–9.2 × 2.8–3.9 µm. *Peniophorella crassibasidiata*, belonging to *Hymenochaetales*, is characterised by soft coriaceous basidiomata, thick-walled generative hyphae, the presence of three types of cystidia, cylindrical basidiospores measuring 8.4–12.4 × 3.1–4.2 µm. *Peniophorella variegata*, belonging to *Hymenochaetales*, is characterised by farinaceous basidiomata, smooth hymenophoral surface, three types of cystidia, the presence of hyphidia and basidiospores measuring 7.9–10.8 × 3.9–5.1 µm. Phylogenetic analyses of the combined ITS+nLSU dataset using Maximum Likelihood and Bayesian Inference confirmed that one new species belongs to the genus *Botryobasidium (Cantharellales)*, the other two new species belong to the genus *Peniophorella (Hymenochaetales)*, respectively. Phylogenetically related and morphologically similar species to these three new species are discussed.

## Introduction

Wood-inhabiting fungi, mainly belonging to the class *Agaricomycetes (Basidiomycota)*, colonise various woody substrates and play essential roles in decomposing lignin, cellulose, and hemicellulose, thereby driving matter cycles and carbon storage in forest ecosystems (Wang et al. 2013; Dong et al. 2024a, 2025; Hongsanan et al. 2025; Song et al. 2025; Wijesinghe et al. 2025; Yang et al. 2025; Zhao et al. 2025a). Despite their ecological importance and global distribution, the taxonomy of many wood-inhabiting fungal taxa remains poorly known.

The genus *Botryobasidium* Donk belongs to the family *Botryobasidiaceae* Jülich, the order *Cantharellales* Gäum., and is typified by *B. subcoronatum* (Höhn. & Litsch.) Donk. The genus is characterised by annual, resupinate basidiomata with smooth, pellicular, hypochnoid, or arachnoid hymenophores; a monomitic hyphal system; generative mostly branched at right angles; basidia with 2–8 sterigmata; smooth or ornamented basidiospores; and causing a white rot (Donk 1964; Langer et al. 2000b; Moncalvo et al. 2006; Xiong et al. 2009; Zhang et al. 2025). Many asexual morph generic names, such as *Acladium* Link, *Allescheriella* Henn., *Alysidium* Kunze, *Haplotrichum* Link, *Neoacladium* P.N. Singh & S.K. Singh, Physospora Fr., and *Sporocephalium* Chevall., are congeneric with *Botryobasidium*, and were re-combined in *Botryobasidium* (Stalpers et al. 2021). Recent phylogenetic studies based on ITS data or ITS + nLSU data analyses demonstrated that *Botryobasidium* forms a well-supported monophyletic group, consistent with previous evidence from its micromorphological and ultrastructural characteristics, consequently, several new species in the genus have been described in China (Li et al. 2025a, 2025b; Zhang et al. 2025). So far, according to the Index Fungorum (www.indexfungorum.org; accessed on July 10, 2026) and MycoBank (https://www.mycobank.org/page/Simple%20names%20search; accessed on July 10, 2026), 120 and 138 taxa were registered in *Botryobasidium*, respectively, but 97 species are accepted worldwide (Lentz 1967; Jung 1995; Langer et al. 2000a; Hagara 2001; Bernicchia et al. 2010; Saitta et al. 2011; Bates et al. 2017; Kalinina et al. 2020; Ram et al. 2021). Among these accepted species, 24 species were discovered in China (Dong et al. 2024b; Liu et al. 2024; Zhou et al. 2024a; Zhou et al. 2024b; Li et al. 2025a; Wang et al. 2025; Zhang et al. 2025; Yuan et al. 2026).

The genus *Peniophorella* P. Karst. belongs to the family *Peniophorellaceae* L.W. Zhou et al., the order *Hymenochaetales* Oberw., and is typified by *P. pubera* (Fr.) P. Karst. The genus is characterised by annual, effused, thin basidiomata, white to yellowish, smooth to odontioid hymenophores, a monomitic hyphal system, various cystidia, thin-walled, ellipsoid, cylindrical, or allantoid basidiospores with negative reaction in Melzer’s reagent and Cotton Blue, and causing a white rot (Karsten 1889; Bernicchia and Gorjón 2010). The independence of *Peniophorella* was reinstated from *Hyphoderma* Wallr. in hymenochaetoid clade by Larsson (2007a) based on molecular phylogeny, and 19 combinations in *Peniophorella* were proposed. Subsequent studies by Miettinen and Larsson (2011) and Telleria et al. (2012) confirmed that *Peniophorella* forms a well-resolved monophyletic group clearly separated from *Hyphodermasensu stricto*. Although later classified in *Hyphodermataceae* (Justo et al. 2017), more recent studies have proposed the new family *Peniophorellaceae* (Wang et al. 2023), which has been accepted in subsequent works. Meanwhile, based on morphological and molecular phylogenetic analyses, several species have been described in the genus (Dong et al. 2024a; Liu et al. 2024; Zhang et al. 2026). So far, according to the Index Fungorum (www.indexfungorum.org; accessed on July 10, 2026) and MycoBank (https://www.mycobank.org/page/Simple%20names%20search; accessed on July 10, 2026), 46 and 49 specific taxa were registered in *Peniophorella*, respectively, with 46 species accepted worldwide, and 30 species were discovered in China (Larsson 2007b; Duhem 2013; Prasher 2015; Yurchenko et al. 2020; Dong et al. 2024a; Liu et al. 2024; Deng et al. 2025; Li et al. 2025b; Wang et al. 2026; Zhang et al. 2026; Zhu et al. 2026).

A key feature of *Hymenochaetales* is the high diversity of corticioid fungi, which represent a polyphyletic assemblage based on morphology (Liu et al. 2025; Zhao et al. 2025a, 2025b). In the past, the classification of many corticioid genera relied primarily on morphological traits, which often resulted in unnatural groupings. The advent of molecular phylogenetics has substantially improved our understanding of the systematics within these corticioid taxa (Wu et al. 2022; Yuan et al. 2023; Zhou et al. 2023). Several recently established families—such as *Hyphodontiaceae* X.Wei Wang & L.W. Zhou, *Peniophorellaceae*, and *Schizoporaceae* Jülich—mainly consist of corticioid genera, indicating considerable phylogenetic divergence within this morphological group (Wang et al. 2021, 2023). Consequently, combining morphological evidence with phylogenetic analyses remains essential for the accurate delimitation of these inconspicuous fungi.

Currently, the species diversity of *Cantharellales* and *Hymenochaetales* remains largely unexplored, with ongoing studies indicating substantial undiscovered diversity, particularly in biodiverse regions such as East Asia. In the current study, based on ITS + nLSU sequences, one new species belonging to the genus *Botryobasidium (Cantharellales)* and two new species belonging to the genus *Peniophorella (Hymenochaetales)* are described and illustrated.

## Materials and methods

### Morphological studies

Five specimens were collected from natural forests, found on *Pinus massoniana* from Guangdong, Henan and Jiangxi Province of China, respectively. These are deposited in the Fungarium of the Institute of Microbiology, Beijing Forestry University (BJFC), China. Morphological descriptions are based on field notes and voucher specimens. The microscopic analysis follows Spirin et al. (2017) and Wu et al. (2022). Freehand sections were made from dried basidiomata and mounted in 5% (w/v) potassium hydroxide (KOH) to observe colour changes. Sections were studied at a magnification of up to 1000× using a Nikon Eclipse 80i microscope and phase contrast illumination. Microscopic features and measurements were made from slide preparations stained with Cotton Blue and Melzer’s reagent. To represent the variation in the size of spores, 5% of measurements were excluded from each end of the range and are given in parentheses. In the description: KOH = 5% potassium hydroxide, IKI = Melzer’s reagent, IKI– = neither amyloid nor dextrinoid, CB = Cotton Blue, CB– = acyanophilous in Cotton Blue, L = arithmetic average of spore length, W = arithmetic average of spore width, Q = L/W ratios and n = number of basidiospores measured from a given number of specimens. Colour terms follow Anonymous (1969) and Petersen (1996).

### DNA extraction, amplification and sequencing

A CTAB rapid plant genome extraction kit-DN14 (Aidlab Biotechnologies Co., Ltd, Beijing) was used to obtain DNA from dried specimens and to perform the polymerase chain reaction (PCR) according to the manufacturer’s instructions with some modifications (Shen et al. 2019; Sun et al. 2020). The internal transcribed spacer (ITS) and large subunit nuclear ribosomal RNA gene (nLSU) were amplified using the primer pairs ITS5/ITS4 and LR0R/LR7 (White et al. 1990; Hopple and Vilgalys 1999, https://sites.duke.edu/vilgalyslab/rdna_primers_for_fungi/).

PCR amplifications were carried out in 30 μL reaction mixtures containing 15 μL of 2× EasyTaq PCR Super Mix (Beijing Quanshi Gold Biotechnology Co., Ltd., Beijing, China), 12 μL of RNase-free Water, 1 μL of each primer, and 1 μL of genomic DNA. The PCR procedure for ITS was as follows: initial denaturation at 95 °C for 3 min, followed by 34 cycles at 94 °C for 40 s, annealing at 54 °C for 45 s and extension at 72 °C for 1 min, with a final extension of 72 °C for 10 min. The PCR procedure for nLSU was as follows: initial denaturation at 94 °C for 1 min, followed by 34 cycles of denaturation at 94 °C for 30 s, annealing at 50 °C for 1 min and extension at 72 °C for 1.5 min, with a final extension at 72 °C for 10 min. The PCR products were purified and sequenced at the Beijing Genomics Institute (BGI, China), with the same primers. The newlygenerated sequences were deposited in GenBank (Sayers et al. 2024) and listed in Table 1.

**Table 1.** Names, specimen numbers, references and corresponding GenBank accession numbers of the taxa used in the phylogenetic analysis of this study. [New species are in bold; * indicates the type material; — indicates no available sequence].

**Species**	**Sample No**.	**GenBank Accession No**.		**References**
		**ITS**	**nLSU**	
*Botryobasidium acanthosporum*	Yuan16326	PP229497	—	Zhou et al. 2024a
*B. acanthosporum*	Yuan17989	PP229511	—	Zhou et al. 2024a
*B. acanthosporum*	Yuan18083*	PP229512	PP218361	Zhou et al. 2024a
*B. acanthosporum*	Yuan18128	PP229517	—	Zhou et al. 2024a
*B. asperulum*	RAS552	OR471090	OR470959	Swenie et al. 2024
*B. asperulum*	RAS578	OR471100	OR470964	Swenie et al. 2024
*B. aureum*	RAS571 SV1	OR471098	OR470963	Swenie et al. 2024
*B. aureum*	RAS571 SV2	OR471099	—	Swenie et al. 2024
*B. bambusinum*	CLZhao 29916	PQ539057	PQ539060	Dong et al. 2024b
*B. bambusinum*	CLZhao 29936*	PQ539058	PQ539061	Dong et al. 2024b
*B. bambusinum*	CLZhao 29938	PQ539059	PQ539062	Dong et al. 2024b
*B. botryosum*	AFTOL-ID 604	DQ267124	FJ176881	Matheny et al. 2006
*B. candicans*	FRDBI 29580226	OR896129	—	Unpublished
*B. coniferarum*	LWZ20210928-3*	OR557259	OR527282	Liu et al. 2024
*B. coniferarum*	LWZ20171016-15	OR557262	OR527286	Liu et al. 2024
*B. conspersum*	AFTOL-ID 1766	DQ911612	DQ521414	Matheny et al. 2006
*B. daweishanense*	CLZhao 40061	PQ373983	—	Zhang et al. 2025
*B. daweishanense*	CLZhao 40062*	PQ373984	PQ373977	Zhang et al. 2025
*B. gossypirubiginosum*	CLZhao 26052*	OR668924	OR708665	Zhou et al. 2024b
*B. gossypirubiginosum*	Dai 26208	PQ285750	—	Li et al. 2025a
*B. incanum*	CLZhao 26697*	OR668923	OR708664	Zhou et al. 2024b
*B. incanum*	Dai 25375	PQ285751	PQ28566	Li et al. 2025a
*B. indicum*	Yuan18434	PP209217	PP218365	Li et al. 2025a
*B. indicum*	hr5326	OP806032	—	Li et al. 2025a
*B. intertextum*	UC2022959	KP814540	—	Rosenthal et al. 2017
*B. leave*	RAS762	OR471128	—	Swenie et al. 2024
*B. latihyphum*	Dai 26858*	PQ279526	PQ282521	Li et al. 2025a
*B. latihyphum*	Yuan 16496	PP331854	PP218153	Li et al. 2025a
*B. leptocystidiatum*	Yuan17706	PP209200	PP218353	Zhou et al. 2024a
*B. leptocystidiatum*	Yuan17708*	PP209197	PP218354	Zhou et al. 2024a
***B. massonianae***	Dai 35993*	**PZ459029**	**PZ459034**	**Present study**
***B. massonianae***	**Dai 36002**	**PZ459030**	**PZ459035**	**Present study**
*B. robustius*	CBS:945.69	MH859491	MH871272	Vu et al. 2019
*B. robustius*	iNaturalist 162067551	PP436446	—	Unpublished
*B. rubiginosum*	RAS776	OR471136	—	Swenie et al. 2024
*B. simile*	RAS793	OR471146	—	Swenie et al. 2024
*B. simile*	RAS794	OR471147	—	Swenie et al. 2024
*B. subcoronatum*	RAS620 SV1	OR471110	OR470967	Swenie et al. 2024
*B. subincanum*	LWZ20230417-17b	PP959661	PP959649	Wang et al. 2025
*B. subincanum*	LWZ20230417-41a	PP959660	—	Wang et al. 2025
*B. subovalibasidium*	Yuan16439	PP209199	PP218152	Zhou et al. 2024a
*B. subovalibasidium*	Yuan18179*	PP209196	PP218362	Zhou et al. 2024a
*B. tubulicystidium*	DK14_139	OL436769	—	Zhou et al. 2024b
*B. vagum*	LWZ20191016-22	PP959659	PP959648	Wang et al. 2025
*B. xizangense*	LWZ20230722-25a*	PP959663	PP959650	Wang et al. 2025
*B. xizangense*	LWZ20230722-16a	PP959662	—	Wang et al. 2025
*B. yunnanense*	CLZhao 24877*	OR668925	OR708666	Zhou et al. 2024b
*B. zhejiangensis*	Dai 25056*	PQ279530	PQ282525	Li et al. 2025a
*B. zhejiangensis*	Dai 24851	PQ279529	PQ282524	Li et al. 2025a
*Lyomyces allantosporus*	FR 0249548	NR154135	—	Yurchenko et al. 2017
*L. pruni*	GEL2327	DQ340312	—	Yurchenko et al. 2020
*Peniophorella alba*	Yuan 17692*	PV883103	—	Zhu et al. 2026
*P. albohymenia*	CLZhao 33187*	PQ811412	PQ847496	Deng et al. 2025
*P. albohymenia*	CLZhao 33257	PQ811413	—	Deng et al. 2025
*P. aspersa*	TNM F20163	MN062098	MN062143	Yurchenko et al. 2020
*P. aspersa*	TNM F24809*	MN062097	MN062142	Yurchenko et al. 2020
*P. cremea*	CLZhao 1606*	MT955162	—	Xu et al. 2020
*P. cremea*	CLZhao 1719	MT955163	—	Xu et al. 2020
*P. crystallifera*	TNM F23666	KX427170	MN062145	Yurchenko et al. 2020
*P. crystallifera*	LWZ 20210626-4a	ON063685	ON063885	Yurchenko et al. 2020
*P. daweishanensis*	CLZhao 18600*	OR094501	OR449932	Dong et al. 2024a
*P. echinocystis*	KHL 6284	DQ677494	DQ681200	Larsson 2007b
*P. euryhypha*	Dai 25368*	PQ726884	PQ726867	Li et al. 2025b
*P. fissurata*	CLZhao 4539	MN864259	OM985775	Guan et al. 2020
*P. fissurata*	CLZhao 9421*	MN864260	OM985776	Guan et al. 2020
*P. guttulifera*	CBS 107303	LT603016	LT603001	Kolařík and Vohník 2018
*P. guttulifera*	NH 12012	DQ647501	—	Hallenberg et al. 2007
*P. odontiiformis*	MUCL 32673	DQ647498	—	Hallenberg et al. 2007
*P. odontiiformis*	SFC20191015-02	OQ996171	OQ996202	Cho et al. 2024
*P. olivacea*	CLZhao 25896*	OR094502	OR449933	Dong et al. 2024a
*P. pertenuis*	FCUG 2430	DQ647482	—	Hallenberg et al. 2007
*P. pertenuis*	Wu 9606-13	DQ647477	—	Hallenberg et al. 2007
*P. pinicola*	Dai 28400*	PQ726882	PQ726865	Li et al. 2025b
*P. pinicola*	Dai 28468	PQ726883	PQ726866	Li et al. 2025b
*P. pinicola*	Yuan 1444	PQ726889	PQ726872	Li et al. 2025b
*P. praetermissa*	NH 10986	DQ647462	—	Hallenberg et al. 2007
*P. praetermissa*	NH 11192	DQ647461	—	Hallenberg et al. 2007
*P. praetermissa*	Yuan 282	PQ726879	PQ726862	Li et al. 2025b
*P. praetermissa*	Yuan 339	PQ726880	PQ726863	Li et al. 2025b
*P. pubera*	CBS 464 86	MH861988	MH873680	Vu et al. 2019
*P. pubera*	LWZ 20210624-16b	ON063687	ON063887	Wang et al. 2023
*P. punctata*	CLZhao 33720	PQ811414	PQ847497	Deng et al. 2025
*P. punctata*	CLZhao 33732*	PQ811415	PQ847498	Deng et al. 2025
*P. reticulata*	CLZhao 17096	OM985745	OM985783	Unpublished
*P. reticulata*	TNM F22559*	NR172776	NG073752	Yurchenko et al. 2020
*P. rude*	LWZ 20171026-7	ON063688	ON063888	Wang et al. 2023
*P. sidera*	LWZ 20180921-15*	OQ540892	OQ540850	Wang et al. 2023
*P. sidera*	LWZ 20180922-62	OQ540893	OQ540851	Wang et al. 2023
*P. stellata*	Dai 25341	PQ726876	PQ726859	Li et al. 2025b
*P. stellata*	Dai 25358*	PQ726877	PQ726860	Li et al. 2025b
*P. stellata*	Dai 25361	PQ726878	PQ726861	Li et al. 2025b
*P. subalbohymenia*	QYZhang 141*	PX270289	PX270293	Zhang et al. 2026
*P. subalbohymenia*	QYZhang 191	PX270290	PX270294	Zhang et al. 2026
*P. subglobospora*	10628MD	HG315519	—	Telleria et al. 2013
*P. subpraetermissa*	Wu 9506-27*	DQ647493	—	Hallenberg et al. 2007
*P. subreticulata*	LWZ 20200812-37a	OQ540886	OQ540846	Wang et al. 2023
*P. subreticulata*	LWZ 20200921-49b*	OQ540884	OQ540845	Wang et al. 2023
*P. tenuissima*	HMZhou 377*	PV475574	PV646276	Wang et al. 2026
*P. tongbiguanensis*	CLZhao 36353*	PV475575	PV646277	Wang et al. 2026
*P. tsugae*	NH 7473	—	DQ677505	Larsson 2007a
*P. yunnanensis*	CLZhao 4810	MN864263	OM985788	Guan et al. 2020
*P. yunnanensis*	CLZhao 6132	MN864268	OM985789	Guan et al. 2020
***P. crassibasidiata***	Yuan 3208*	**PZ459031**	**PZ459036**	**Present study**
***P. variegata***	Yuan 2232*	**PZ459032**	**PZ459037**	**Present study**
***P. variegata***	**Yuan 2250**	**PZ459033**	**PZ459038**	**Present study**
*Schizocorticium magnosporum*	Wu 1510-34	MK405351	MK405337	Wu et al. 2021
*S. mediosporum*	Chen 2456	MK405359	MK405345	Wu et al. 2021

Sequences generated were aligned manually with additional sequences downloaded from GenBank using AliView version 1.27 (Larsson 2014). The final ITS and nLSU datasets were subsequently aligned using MAFFT v.7 under the E-INS-i strategy with no cost for opening gaps and equal cost for transformations (command line: mafft –genafpair –maxiterate 1000, Katoh and Standley (2013) and visualised in AliView. Alignments were spliced and transformed formats in Mesquite v.3.2. (Maddison and Maddison 2017). Multiple sequence alignments were trimmed by trimal v.1.2 using the -htmlout-gt 0.8 -st option to deal with gaps, when necessary (Capella-Gutierrez et al. 2009).

### Phylogenetic analyses

**Figure 1.**
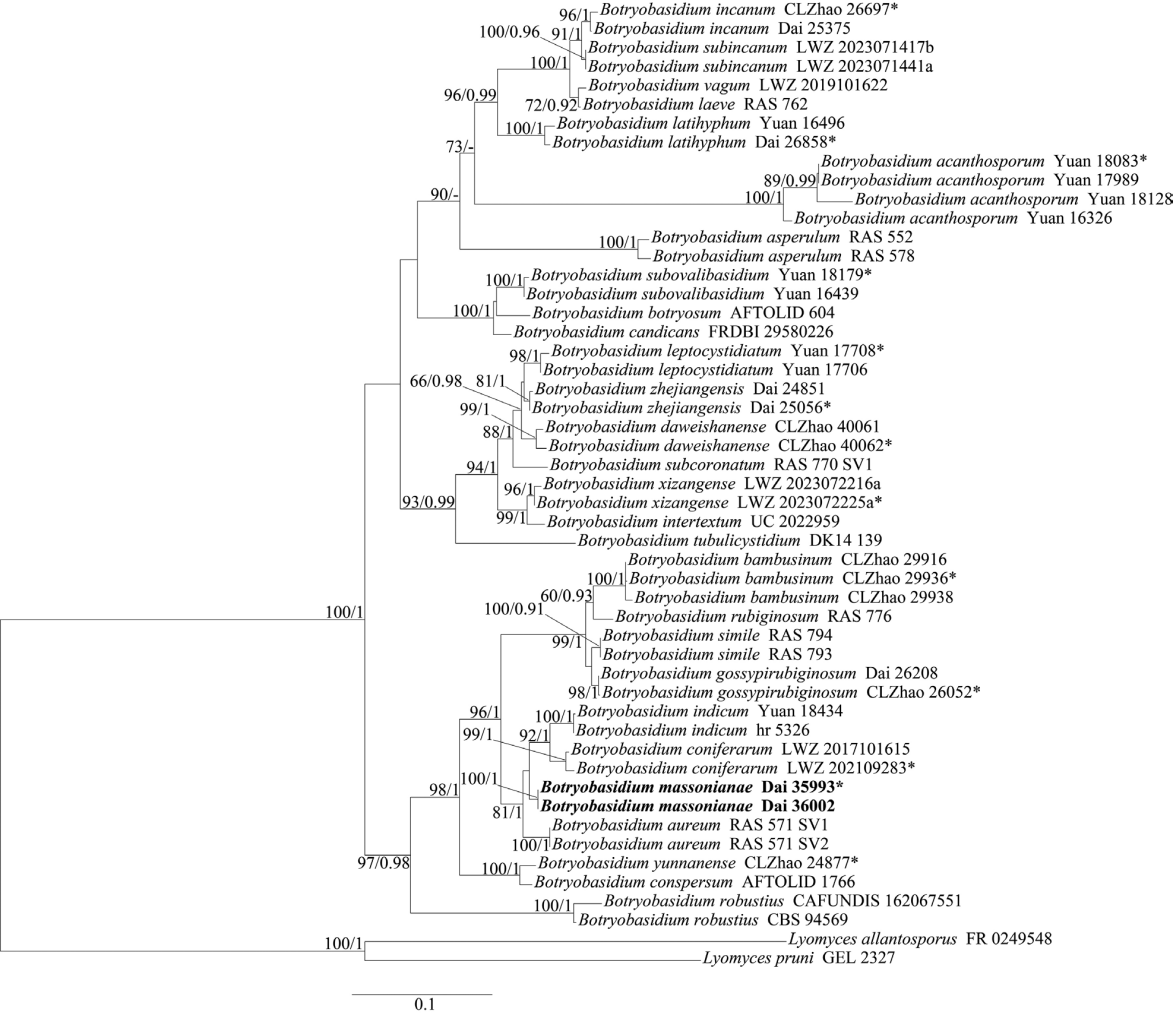
ML analysis of *Botryobasidium*, based on dataset of ITS + nLSU. ML bootstrap values equal to or higher than 60% and Bayesian posterior probabilities values equal to or higher than 0.90 are shown. New taxa are in bold, * represents type material.

**Figure 2.**
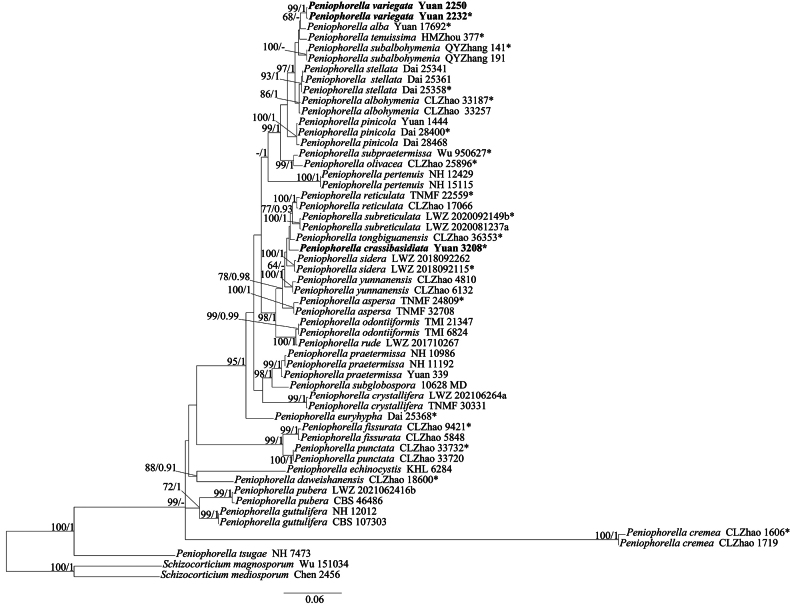
ML analysis of *Peniophorella*, based on dataset of ITS + nLSU. ML bootstrap values equal to or higher than 60% and Bayesian posterior probabilities values equal to or higher than 0.90 are shown. New taxa are in bold, * represents type material.

The two-marker DNA multiple sequence alignment (ITS + nLSU) was used to determine the phylogenetic position of the new species. Sequences of *Lyomyces allantosporus* Riebesehl, Yurchenko & Langer and *L. pruni* (Lasch) Riebesehl & Langer were chosen as the outgroups (Fig. 1, Spirin et al. 2015; Riebesehl and Langer 2017; Chen and Zhao 2020). Sequences of *Schizocorticium magnosporum* Sheng H. Wu & C.L. Wei and *S. mediosporum* Sheng H. Wu & C.L. Wei were chosen as the outgroups (Fig. 2, Cho et al. 2024). The phylogenetic analyses followed the approach of Han et al. (2015) and Zhu et al. (2019). Maximum Likelihood (ML) and Bayesian Inference (BI) analyses were performed, based on the ITS + nLSU datasets.

Sequences were analysed using Maximum Likelihood (ML) with RAxML v.8.2.10 (Stamatakis 2014). Branch supports for all parsimony analyses were estimated by performing 1,000 bootstrap replicates with a heuristic search of 10 random-addition replicates for each bootstrap replicate. Bayesian phylogenetic inference and Bayesian Posterior Probabilities (BPP) were computed with MrBayes 3.2.6 with a GTR + I + G model of DNA substitution and a gamma distribution rate variation across sites (Ronquist and Huelsenbeck 2003). Four Markov chains were run for 1.5 million generations (two-marker dataset) until the split deviation frequency value was less than 0.01 and trees were sampled every 100 generations in Fig. 1; for 2 million generations in Fig. 2. The first 25% of the sampled trees were discarded as burn-in and the remaining ones were used to reconstruct a majority rule consensus and calculate Bayesian Posterior Probabilities (BPP) of the clades. All trees were viewed in FigTree v. 1.4.3 (http://tree.bio.ed.ac.uk/software/figtree/). Branches that received bootstrap support for ML (≥ 75% (ML-BS)) and BPP (≥ 0.95 BPP) were considered as significantly supported. The ML bootstrap (ML) ≥ 50% and Bayesian Posterior Probabilities (BPP) ≥ 0.90 are presented on topologies from ML analysis, respectively.

## Results

### Molecular phylogeny

The combined two-marker dataset (ITS + nLSU, Fig. 1) included sequences from 51 samples representing 30 taxa. The phylogenetic reconstruction performed with Maximum Likelihood (ML) and Bayesian Inference (BI) analyses for the combined dataset showed similar topology and few differences in statistical support. The best model-fit applied in the Bayesian analysis was GTR + I + G, lset nst = 6, rates = invgamma and prset statefreqpr = dirichlet (1, 1, 1, 1). Bayesian analysis and ML analysis resulted in a similar topology to the MP analysis, with an average standard deviation of split frequencies of 0.007700 (BI). The phylogenetic tree inferred from the ITS + nLSU sequences indicated that the new species belonged to *Botryobasidium* (Fig. 1). In addition, *Botryobasidium massonianae* grouped together with *B. aureum* Parmasto, *B. coniferarum* S.L. Liu & L.W. Zhou and *B. indicum* (P.N. Singh & S.K. Singh) R. Kirschner & G. Langer with high support (ML = 81%, BPP = 1.00).

The combined two-marker dataset (ITS + nLSU, Fig. 2) included sequences from 55 samples representing 33 taxa. The phylogenetic reconstruction performed with Maximum Likelihood (ML) and Bayesian Inference (BI) analyses for the combined dataset showed similar topology and few differences in statistical support. The best model-fit applied in the Bayesian analysis was GTR + I + G, lset nst = 6, rates = invgamma and prset statefreqpr = dirichlet (1, 1, 1, 1). Bayesian analysis and ML analysis resulted in a similar topology to the MP analysis, with an average standard deviation of split frequencies of 0.008584 (BI). The phylogenetic tree inferred from the ITS + nLSU sequences indicated that the two new species belonged to *Peniophorella* (Fig. 2). In addition, *Peniophorella crassibasidiata* formed an independent lineage; *P. variegata* is related to *P. alba* L.J. Zhou et al., *P. albohymenia* Y.L. Deng & C.L. Zhao, *P. stellata* Y.C. Dai et al., *P. subalbohymenia* Q.Y. Zhang, and *P. tenuissima* Lu Wang & C.L. Zhao with high support (ML = 97%, BPP = 1.00).

Based on the ITS and nLSU sequences of the type material, BLAST queries against the authoritative sequences in NCBI yielded alignments, which are shown in Table 2.

**Table 2.** Based on the ITS and nLSU sequences of the type material, BLAST queries against the authoritative sequences in NCBI yielded alignments.

**Species name**	**Marker**	**Top match species**	**GenBank Accession No**.	**Max score**	**Total score**	**Query cover**	**E-value**	**Identity**
*Botryobasidium massonianae*	ITS	*B. indicum*	PV191125	1002	1002	99%	0.0	94.74%
	nLSU	*B. aureum*	OR470963	2370	2370	99%	0.0	98.37%
*Peniophorella crassibasidiata*	ITS	*P. reticulata*	NR172776	1064	1064	96%	0.0	96.31%
	nLSU	*P. aspersa*	OM985770	1797	1797	99%	0.0	99.69%
*P. variegata*	ITS	*P. subalbohymenia*	PX270289	1118	1118	99%	0.0	96.35%
	nLSU	*P. subpraetermissa*	OM985785	2431	2431	99%	0.0	99.4%

### Taxonomy

#### Botryobasidium massonianae

Taxon classificationFungiCantharellalesBotryobasidiaceae

K.Y. Luo, Yuan Yuan & Y.C. Dai sp. nov.

13338FD3-5060-5669-AEAB-DA8D6E3E8FCE

864541

Figs 3, 4

##### Holotype.

China • Henan Province, Xinyang, Shangcheng County, Huangbaishan Forest Park. GPS coordinates: 31.459192°N, 115.370512°E; elevation: 500 m a.s.l. On a fallen branch of *Pinus massoniana*, 20 September 2025, Dai 35993 (BJFC 057254).

**Figure 3.**
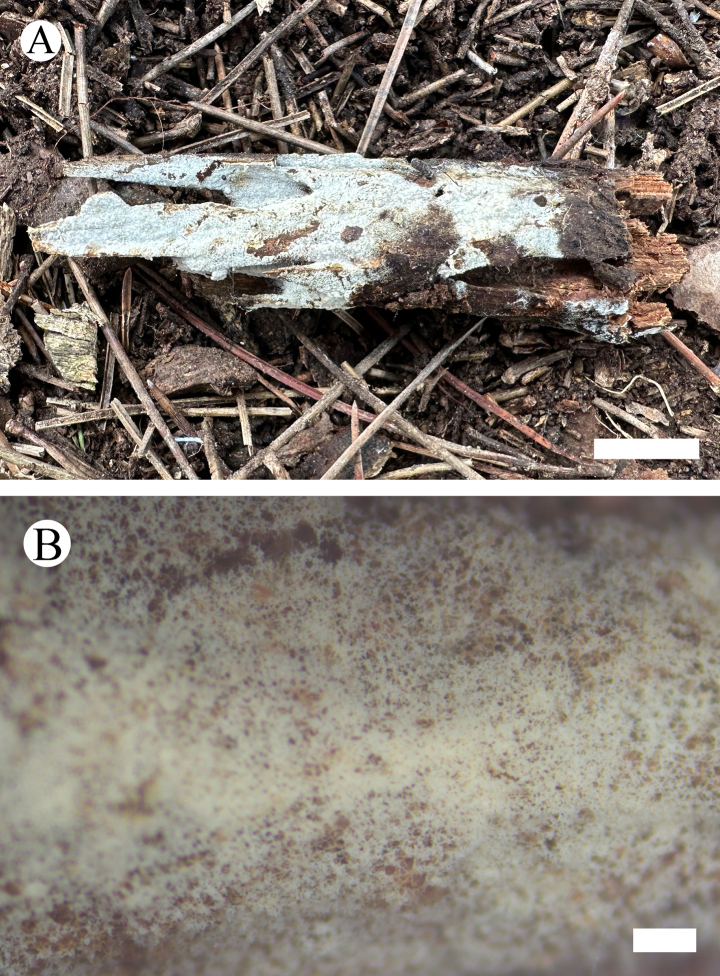
Basidiomata of *Botryobasidium massonianae* (Holotype, Dai 35993). Scale bar: 1 cm (**A**); 0.3 mm (**B**).

**Figure 4.**
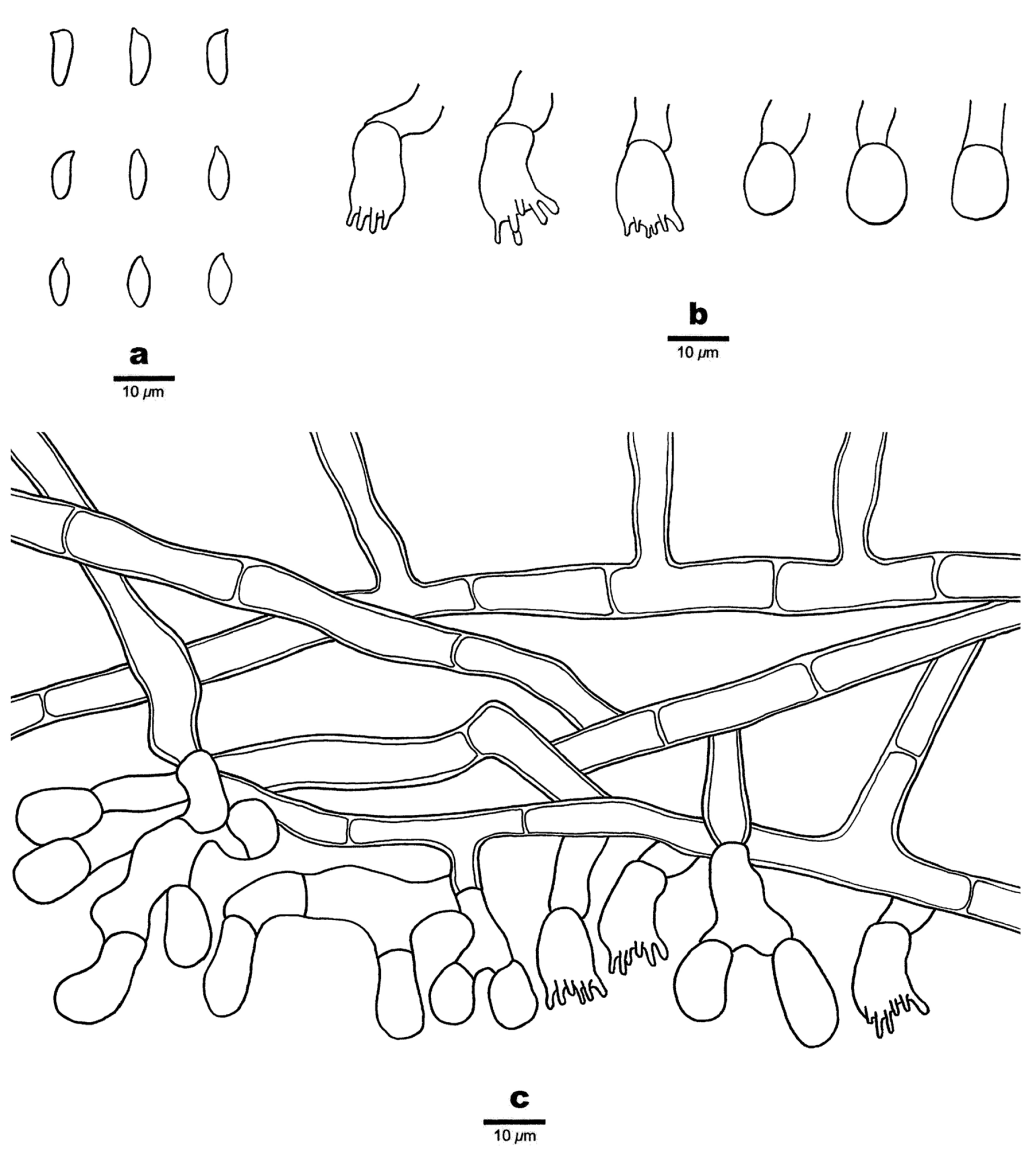
Microscopic structures of *Botryobasidium massonianae* (Holotype, Dai 35993). **a**. Basidiospores; **b**. Basidia and basidioles; **c**. A section of the basidioma.

##### Etymology.

*Massonianae* (Lat.) refers to the species growing on *Pinus massoniana*.

##### Description.

Basidiomata annual, resupinate, effuse, fluffy, pellicular, adherent to the substrate and not easily separated, without odour or taste, up to 6 cm long, 1.5 cm wide, and 200 µm thick at centre. Hymenophoral surface hypochnoid, white when fresh, cream when dry. Sterile margin often indeterminate and not differentiated.

Hyphal structure: Hyphal system monomitic; generative hyphae simple septate, slightly thick-walled with wide lumen, moderately branched mostly with a right angle, more or less straight, loosely interwoven, 5–7.5 μm in diam; IKI–, CB–; tissues unchanged in KOH.

Hymenium: Cystidia and cystidioles absent. Basidia barrel-shaped, thin-walled, with four to six sterigmata and a basal simple septum, 11–16 × 5–10 µm; basidioles in shape similar to basidia, but slightly shorter.

Basidiospores: Fusiform, hyaline, thin-walled, smooth, IKI–, CB–, (5.7–)6.3–9.2(–10.2) × (2.7–)2.8–3.9(–4.3) µm, L = 7.46 µm, W = 3.31 µm, Q = 2.24–2.26 (n = 60/2).

##### Type of rot.

White rot.

##### Additional specimen (paratype) examined.

China • Henan Province, Xinyang, Shangcheng County, Huangbaishan Forest Park. GPS coordinates: 31.459192°N, 115.370512°E; elevation: 500 m a.s.l. On a fallen branch of *Pinus massoniana*, 20 September 2025, Dai 36002 (BJFC 057263).

#### Peniophorella crassibasidiata

Taxon classificationFungiHymenochaetalesRickenellaceae

K.Y. Luo, Yuan Yuan & Y.C. Dai sp. nov.

0D622D0E-908F-506C-AB05-F74BC3263983

864542

Figs 5, 6

##### Holotype.

China • Guangdong Province, Zhaoqing, Dinghu District, Dinghushan National Nature Reserve. GPS coordinates: 23.159571°N, 112.538344°E; elevation: 51 m a.s.l. On a fallen branch of *Pinus massoniana*, 8 July 2025, Yuan 3208 (BJFC 062754).

**Figure 5.**
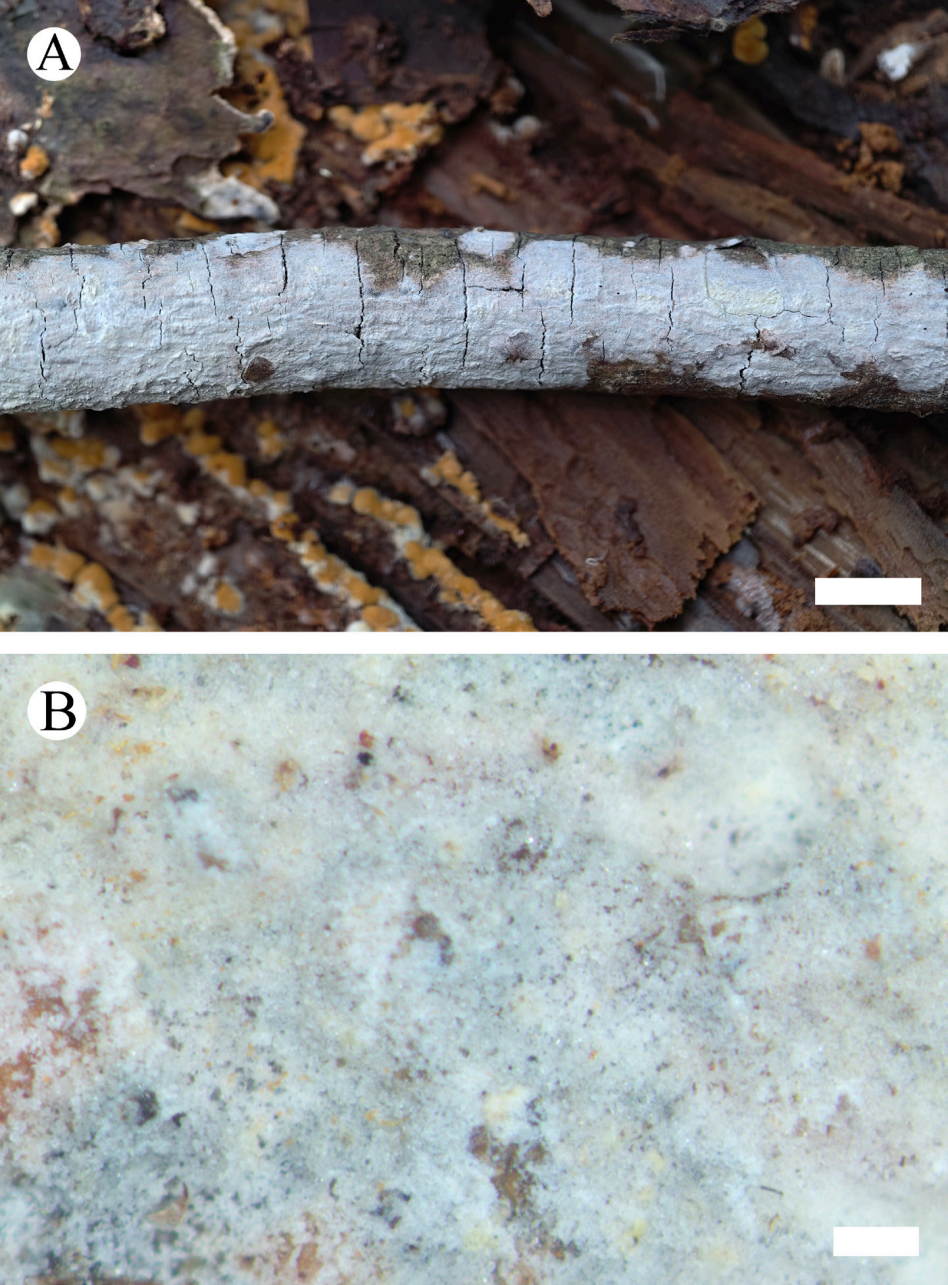
Basidiomata of *Peniophorella crassibasidiata* (Holotype, Yuan 3208). Scale bar: 1 cm (**A**); 0.2 mm (**B**).

**Figure 6.**
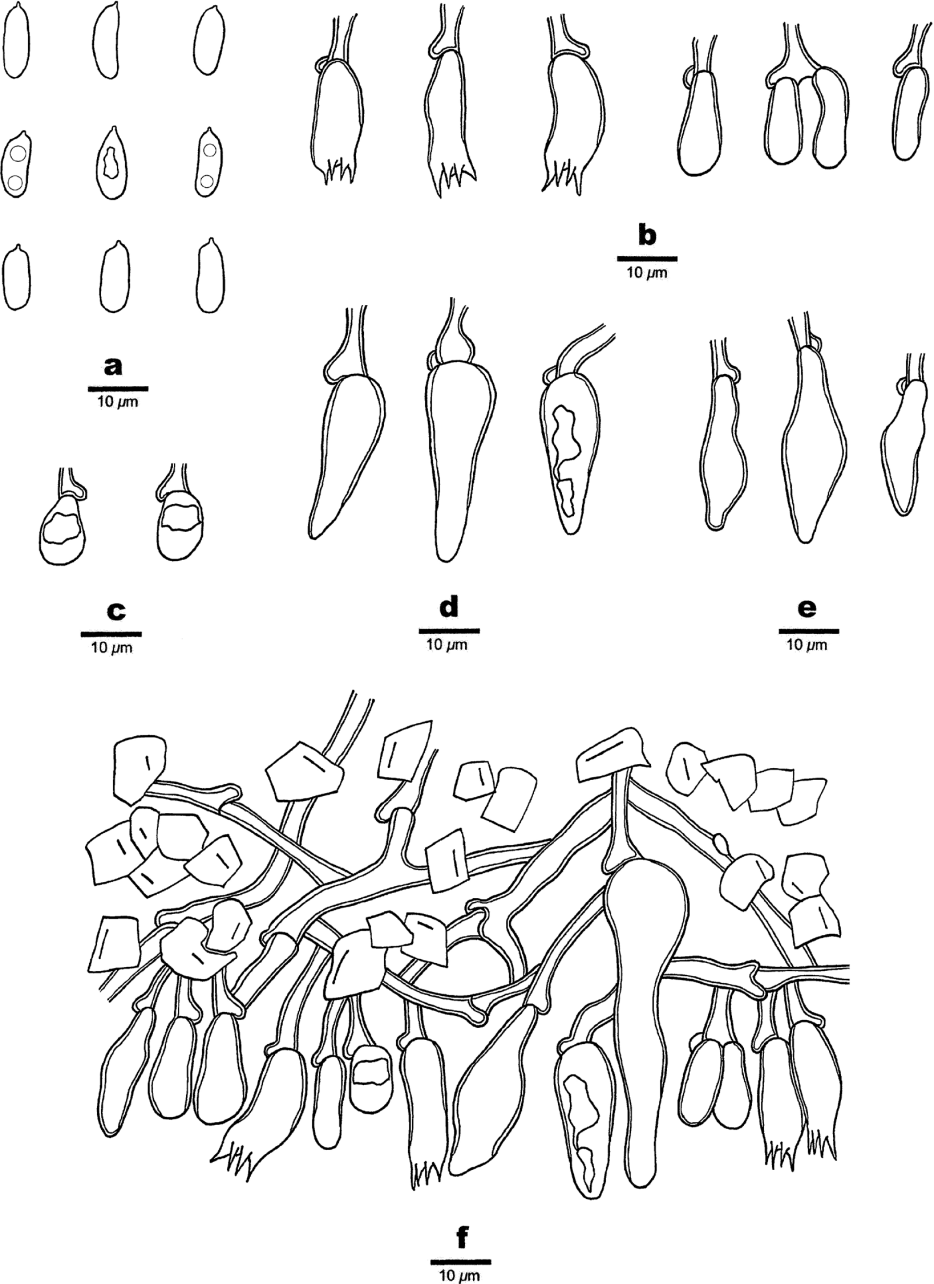
Microscopic structures of *Peniophorella crassibasidiata* (Holotype, Yuan 3208). **a**. Basidiospores; **b**. Basidia and basidioles; **c**. Stephanocysts; **d**. Subulate cystidia; **e**. Fusiform cystidia; **f**. A section of the basidioma.

##### Etymology.

*Crassibasidiata* (Lat.), crassi- = thick, basidiata = basidia, referring to the thick-walled basidia of this species.

##### Description.

Basidiomata annual, resupinate, adnate, loosely attached, but not easily separable from substratum, soft coriaceous, without odor and taste when fresh, white when fresh, white to cream upon drying, up to 14 cm long, 1.5 cm wide, and 200 µm thick at center. Hymenophoral surface smooth; margin distinct, thin, white, up to 2 mm long.

Hyphal structure: Hyphal system monomitic; generative hyphae with clamp connections, thick-walled with a wide lumen, occasionally branched, flexuous, loosely interwoven, 2–4 μm in diameter, IKI–, CB–; tissues unchanged in KOH.

Hymenium: Cystidia of three types: (1) stephanocysts infrequent, with some oily contents, hyaline, thin-walled, 9–11 × 6–7 μm; (2) fusiform cystidia frequent, hyaline, thick-walled, 22–25 × 7–9 μm; (3) subulate cystidia frequent, sometimes with oily contents, hyaline, thick-walled, 20–50 × 6–10 μm. Cystidioles absent. Nubbly crystals abundant. Basidia barrel-shaped to subclavate, colorless, thick-walled, smooth, with four sterigmata and a basal clamp connection, 12–22 × 5–9 μm. Basidioles similar in shape to basidia, but smaller.

Basidiospores: cylindrical, hyaline, thin-walled, smooth, occasionally with two oil drops or contents, IKI–, CB–, (8–)8.4–12.4(–12.6) × (3–)3.1–4.2(–4.4) µm, L = 10.21 µm, W = 3.67 µm, Q = 2.78 (n = 30/1).

##### Type of rot.

White rot.

#### Peniophorella variegata

Taxon classificationFungiHymenochaetalesRickenellaceae

K.Y. Luo, Yuan Yuan & Y.C. Dai sp. nov.

30CE719D-A320-5C85-86CA-33E20379D517

864543

Figs 7, 8

##### Holotype.

China • Jiangxi Province, Nanchang, Shengshuitang National Forest Park. GPS coordinates: 28.991150°N, 115.533628°E; elevation: 220 m a.s.l. On a fallen branch of *Pinus massoniana*, 26 April 2025, Yuan 2232 (BJFC 061778).

**Figure 7.**
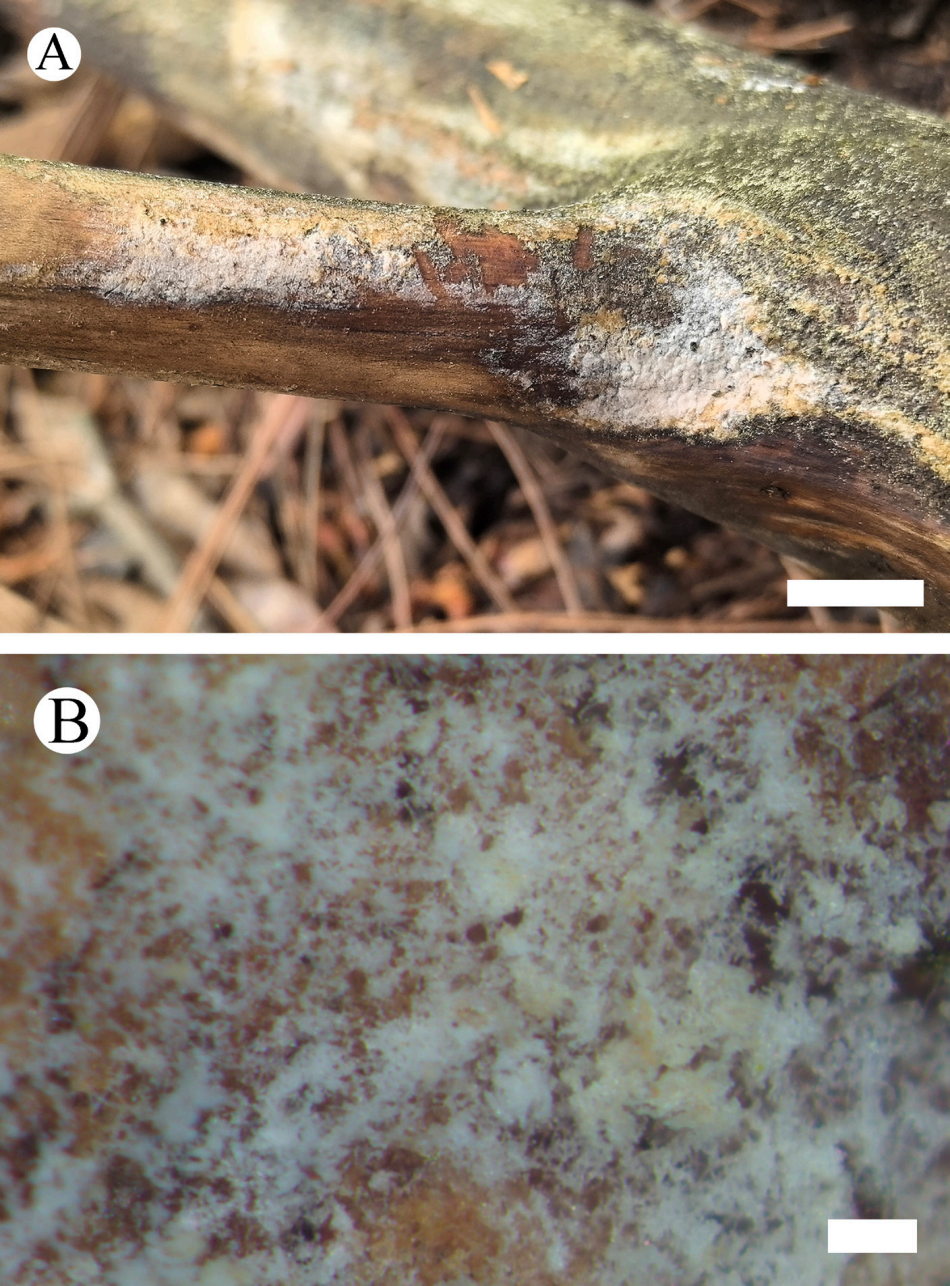
Basidiomata of *Peniophorella variegata* (Holotype, Yuan 2232). Scale bar: 1 cm (**A**); 0.1 mm (**B**).

**Figure 8.**
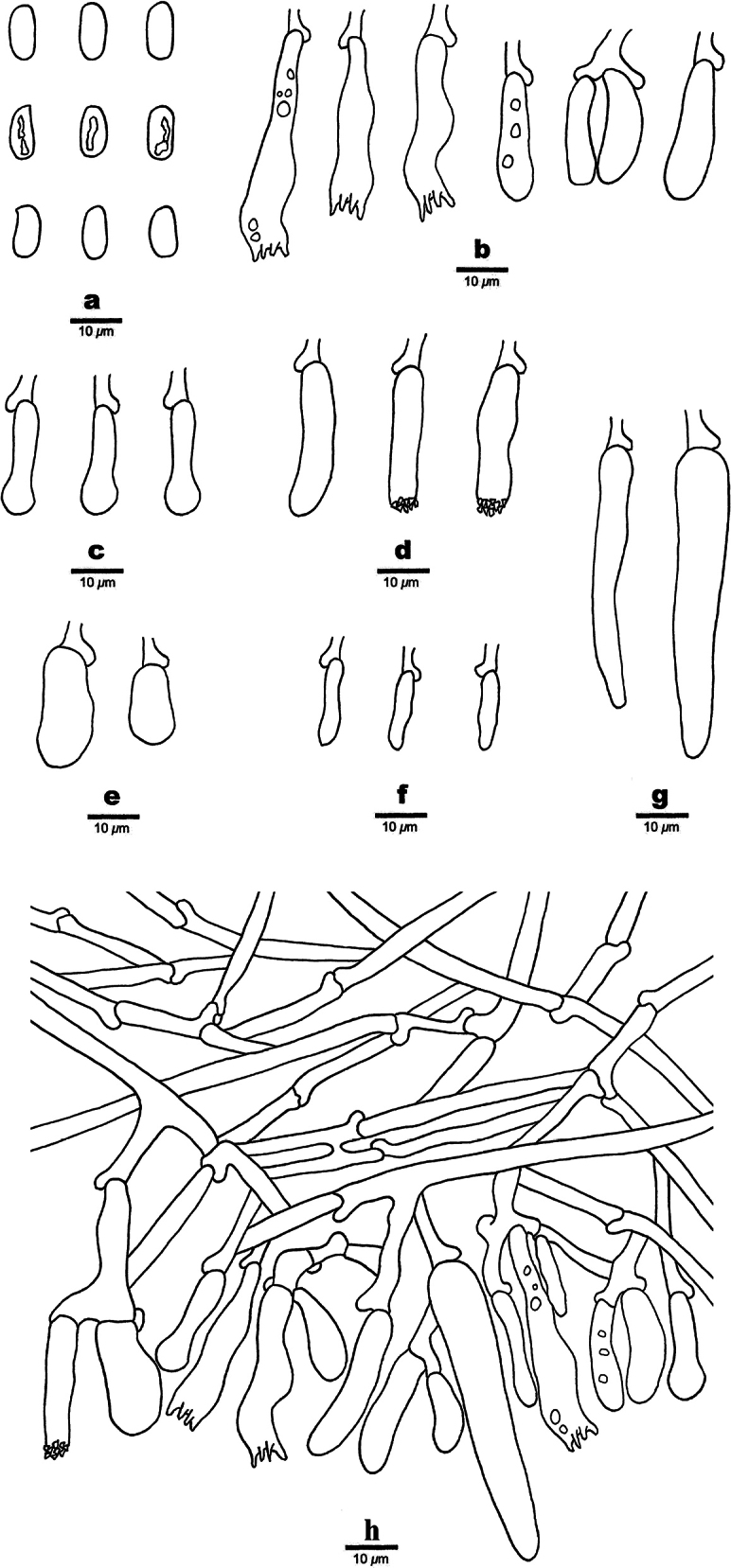
Microscopic structures of *Peniophorella variegata* (Holotype, Yuan 2232). **a**. Basidiospores; **b**. Basidia and basidioles; **c**. Capitate cystidia; **d**. Leptocystidia; **e**. Cystidioles; **f**. Hyphidia; **g**. Subulate cystidia; **h**. A section of the basidioma.

##### Etymology.

*Variegata* (Lat.): refers to the species having variable cystidia.

##### Description.

Basidiomata annual, resupinate, adnate, loosely attached, not easily separable from substratum, farinaceous, without odour and taste when fresh, white to pale yellowish when fresh and upon drying, up to 7 cm long, 1.5 cm wide, and 300 µm thick. Hymenophoral surface smooth; margin distinct, thin, pale yellowish, up to 2 mm long.

Hyphal structure: Hyphal system monomitic; generative hyphae with clamp connections, thin-walled, moderately branched mostly with a right angle, flexuous, loosely interwoven, 2.5–4 μm in diameter, IKI–, CB–; tissues unchanged in KOH.

Hymenium: Cystidia of three types: (1) capitate cystidia frequent, hyaline, thin-walled, 18.5–24 × 4–7 μm; (2) leptocystidia infrequent, subcylindrical, hyaline, thin-walled, sometimes apically encrusted, 19–31 × 5–7 μm; (3) subulate cystidia infrequent, hyaline, thin-walled, sometimes protruding from the hymenium up to 20 μm, 38–53 × 4–7 μm. Hyphidia frequent, hyaline, thin-walled, 13–16 × 2.5–4 μm. Cystidioles rare, subcylindrical or sphaeropedunculate, hyaline, thin-walled, 11–14 × 6–8 μm. Basidia clavate, more or less flexuous, colorless, thin-walled, smooth, with four sterigmata and a basal clamp connection, occasionally with oily contents, 25–46 × 6–12 μm. Basidioles similar in shape to basidia, but distinctly smaller.

Basidiospores: Short cylindric to oblong-ellipsoid, sometime slightly curved, hyaline, thin-walled, smooth, occasionally with oily contents, IKI–, CB–, (7.3–)7.9–10.8(–12.5) × (3.1–)3.9–5.1(–5.8) µm, L = 9.44 µm, W = 4.50 µm, Q = 2.07–2.13 (n = 60/2).

##### Type of rot.

White rot.

##### Additional specimen (paratype) examined.

China • Jiangxi Province, Nanchang, Shengshuitang National Forest Park. GPS coordinates: 28.991150°N, 115.533628°E; elevation: 220 m a.s.l. On a fallen branch of *Pinus massoniana*, 26 April 2025, Yuan 2250 (BJFC 061796).

## Discussion

*Cantharellales* and *Hymenochaetales* are two important orders within the class *Agaricomycetes (Basidiomycota)* that exhibit significant differences in ecological function, wood-inhabiting mechanisms, and phylogenetic relationships. In recent years, with the rapid development of molecular phylogenetic methods and the increasing intensity of global surveys of forest fungal diversity, important progress has been made in understanding the species diversity, taxonomic framework, and evolutionary history of these two orders (Wang et al. 2018; Swenie et al. 2025; Zhao et al. 2025a; Kumar et al. 2026).

*Cantharellales* is an ecologically highly diverse order, encompassing saprotrophic, mycorrhizal, lichenised, and parasitic nutritional modes (Tuo et al. 2026; Xu et al. 2026). One new species discovered in this study, *Botryobasidium massonianae*, is a saprotrophic fungus growing on *Pinus massoniana*.

*Hymenochaetales* includes a large number of important wood-decaying fungi including some forest pathogens and medicinal fungi (Dai et al. 2000; Wu et al. 2004; Wu et al. 2019; Wu and Dai 2020), and have a worldwide distribution. This order contains corticioid, poroid and hydnoid fungi with different types of basidiomata (Dai et al. 2004; Wu et al. 2022; Zhou et al. 2023; Zhao et al. 2025a). The two species *Peniophorella crassibasidiata* and *P. variegata* discovered in this study are both corticioid fungi.

In our phylogeny (Fig. 1), *Botryobasidium massonianae* grouped together with *B. aureum*, *B. coniferarum* and *B. indicum* with high support (ML = 81%, BPP = 1.00). However, *B. aureum* is different from *B. massonianae* by its thin-walled generative hyphae (Parmasto 1965); *B. coniferarum* is readily distinguished from *B. massonianae* by longer basidia (16–20 µm vs. 11–16 µm, Liu et al. 2024); *B. indicum* differs from *B. massonianae* by its yellow basidiomata (Hyde et al. 2019). Morphologically, *B. massonianae*, *B. acanthosporum* L.J. Zhou & H.S. Yuan and *B. subovalibasidium* L.J. Zhou & H.S. Yuan share simple septate generative hyphae. However, *B. acanthosporum* is different from *B. massonianae* by its basidia with 2 sterigmata and subglobose to globose, slightly thick- to thick-walled basidiospores; *B. subovalibasidium* differs from *B. massonianae* by its pale yellow to greyish yellow hymenophoral surface (Zhou et al. 2024a).

In our phylogeny (Fig. 2), *Peniophorella crassibasidiata* formed an independent lineage. Morphologically, *P. crassibasidiata*, *P. pinicola* Y.C. Dai et al. and *P. stellata* share the presence of stephanocysts. However, *P. pinicola* is different from *P. crassibasidiata* by its shorter basidiospores (5.7–8 µm vs. 8.4–12.4 µm); *P. stellata* differs from *P. crassibasidiata* by its longer basidia (22.5–32 µm vs. 12–22 µm, Li et al. 2025b).

*Peniophorella variegata* is related to *P. alba*, *P. albohymenia*, *P. stellata*, *P. subalbohymenia*, and *P. tenuissima* with high support (ML = 97%, BPP = 1.00). However, *P. alba* is different from *P. variegata* by its thick-walled cystidia (Zhu et al. 2026); *P. albohymenia* is readily distinguished from *P. variegata* by its absence of hyphidia and cystidioles (Deng et al. 2025); *P. stellata* differs from *P. variegata* a (Li et al. 2025b); *P. subalbohymenia* is different from *P. variegata* by its shorter basidia (18–25 µm vs. 25–46 µm, Zhang et al. 2026); *P. tenuissima* is readily distinguished from *P. variegata* by its wider basidiospores (5.5–7.5 µm vs. 3.9–5.1 µm, Wang et al. 2026). Morphologically, *P. variegata*, *P. olivacea* J.H. Dong & C.L. Zhao and *P. tongbiguanensis* L. Wang & C.L. Zhao share farinaceous basidiomata. However, *P. olivacea* differs from *P. variegata* by its presence of halocystidia, the absence of hyphidia, and both shorter basidia (17.5–20 µm vs. 25–46 µm) and basidiospores (6.5–7.5 µm vs. 7.9–10.8 µm, Dong et al. 2024a); *P. tongbiguanensis* is different from *P. variegata* by lacking capitate cystidia, hyphidia and cystidioles, and having larger basidiospores (11–13.5 × 5.5–7 µm vs. 7.9–10.8 × 3.9–5.1 µm, Wang et al. 2026).

We collected specimens from Guangdong, Henan, and Jiangxi provinces, representing southern, central, and eastern China, respectively. Notably, new species belonging to these two orders were discovered in all these areas, indicating that the diversity of *Cantharellales* and *Hymenochaetales* is more widely distributed across different climatic zones of China. This broader geographic coverage strengthens the significance of our findings and provides a more comprehensive perspective on the national diversity of these fungal groups.

## Supplementary Material

XML Treatment for Botryobasidium massonianae

XML Treatment for Peniophorella crassibasidiata

XML Treatment for Peniophorella variegata

## Additional information

### Artificial Intelligence (AI) use

The authors accept full responsibility for the content of the manuscript, including the disclosure of any use of AI.

No AI tools were used in the preparation of this manuscript.

### Author ORCIDs

K.-Y. Luo https://orcid.org/0000-0001-6145-4864

Y.-C. Dai https://orcid.org/0000-0002-6523-0320

Y. Yuan https://orcid.org/0000-0001-6674-9848

Y.-D. Wu https://orcid.org/0000-0003-1295-4015

### Data availability

All of the data that support the findings of this study are available in the main text.
